# The Pleiotropic Effects of Glutamine Metabolism in Cancer

**DOI:** 10.3390/cancers11060770

**Published:** 2019-06-04

**Authors:** Alex J. Bott, Sara Maimouni, Wei-Xing Zong

**Affiliations:** 1 Department of Biochemistry, University of Utah School of Medicine, Salt Lake City, UT 84112, USA; 2 Department of Chemical Biology, Ernest Mario School of Pharmacy, Rutgers Cancer Institute of New Jersey, Rutgers-The State University of New Jersey, 164 Frelinghuysen Rd, Piscataway, NJ 08854, USA

**Keywords:** glutamine, glutamate, glutaminolysis, glutaminase (GLS), glutamate ammonia ligase (GLUL), glutamine synthetase (GS), cancer, metabolism

## Abstract

Metabolic programs are known to be altered in cancers arising from various tissues. Malignant transformation can alter signaling pathways related to metabolism and increase the demand for both energy and biomass for the proliferating cancerous cells. This scenario is further complexed by the crosstalk between transformed cells and the microenvironment. One of the most common metabolic alterations, which occurs in many tissues and in the context of multiple oncogenic drivers, is the increased demand for the amino acid glutamine. Many studies have attributed this increased demand for glutamine to the carbon backbone and its role in the tricarboxylic acid (TCA) cycle anaplerosis. However, an increasing number of studies are now emphasizing the importance of glutamine functioning as a proteogenic building block, a nitrogen donor and carrier, an exchanger for import of other amino acids, and a signaling molecule. Herein, we highlight the recent literature on glutamine’s versatile role in cancer, with a focus on nitrogen metabolism, and therapeutic implications of glutamine metabolism in cancer.

## 1. Introduction

Observations of altered cancer cell metabolism date to the 1920s, in which Warburg observed that the preference of rapidly dividing cancer cells to undergo glycolysis, even in an oxygen replete environment (or “aerobic glycolysis”, Warburg Effect) [1]. This alteration in metabolic substrate utilization has been highlighted as an emerging hallmark of cancer, and can be observed in disease regardless of the driving oncogene or tissue of origin [2]. Alongside changes in glucose metabolism, cancer cells have shown extensive metabolic flexibility including altered lipid metabolism, mitochondrial metabolism, and amino acid metabolism [3,4,5,6,7,8,9]. Notable among amino acids is increased usage of glutamine.

Glutamine is the most abundant amino acid in plasma [10,11]. The majority of glutamine in circulation is produced in muscles, with the lung as an additional source [12,13]. By definition, glutamine is a non-essential amino acid but has been noted to be “conditionally essential” during pathological stress such as pathogen infection and starvation [14,15]. Consequently, clinical studies have suggested glutamine supplementation can reduce the mortality rate in burn patients and patients with acute pancreatitis [16,17].

**Figure 1:**
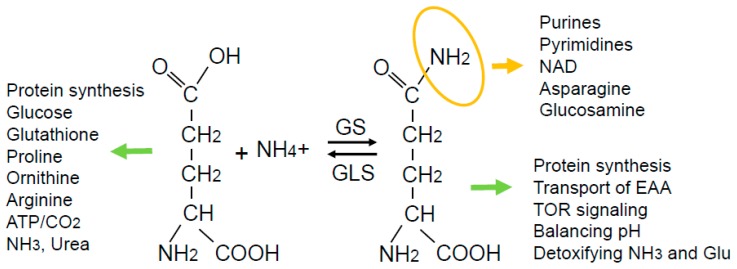
While glutamine can be converted to glutamate to exert functions indicated on the left-hand side, glutamine has its own specific functions: (1) The terminal amide group in glutamine (highlighted in orange) is the primary nitrogen source for several macromolecules; (2) the entire glutamine molecule can function to drive the uptake of essential amino acids (EAA) and activate mTOR; (3) the glutamine synthesis reaction can help balance pH and detoxify ammonia and glutamate.

In addition to its familiar role as a constituent of proteins, glutamine is an anaplerotic substrate for the tricarboxylic acid (TCA) cycle, contributing its carbon backbone particularly in conditions of carbon diversion to glycolytic pathways. It is also the obligatory nitrogen donor for the biosynthesis of purines, pyrimidines, nicotinamide adenine dinucleotide (NAD), asparagine, and hexosamines via its terminal amide group. Glutamine also drives the uptake of essential amino acids, activates mTOR, helps recycle excessive ammonia and glutamate, and regulates redox (Figure 1). Accordingly, it is conceivable that intracellular glutamine needs to be maintained at a critical level, which may be further upregulated in cancer cells. In many cancer cells, glutamine demand dominates supply in periods of rapid growth [18]. The term ‘glutamine addiction’ has been used to describe the enhanced usage of glutamine in cancer, predominately in a catabolic/anaplerotic sense [19].

## 2. Uptake and Exchange

Many tissue types and cancer cells can obtain glutamine from the circulation. A number of transporters have been identified which transport glutamine across the plasma membrane. Among these are members of the solute carrier family (SLC) proteins that contain in excess of 400 members [20]. Glutamine can be transported across the plasma membrane by SLC1A5 and SLC7A5/SLC3A2 [21]. While these transporters can import glutamine for subsequent catabolism and energy production, it has been shown that glutamine imported via the transporters can also promote rapamycin-sensitive mTOR signaling. This is mediated by intracellular glutamine being effluxed from the cell in exchange of the uptake of essential amino acids such as leucine [21]. This use of glutamine as an exchange factor has been further developed, identifying additional roles of glutamine, as well as asparagine, for this function [22]. Interestingly, these transporters are upregulated in cancers arising from a multitude of tissues and under various oncogenic drivers [23,24,25]. The master transcriptional regulator of metabolism, c-Myc, has been shown to directly bind to the upstream promoter of ASCT2 (SLC1A5) to increase its expression [26]. Increased expression of the transporter indeed leads to elevated uptake of glutamine and this is coupled to increased catabolic usage [26]. In prostate cancer, androgen receptor, c-Myc, and mTOR collectively act to increase expression of glutamine transporters to facilitate glutamine uptake and cancer cell growth [25]. In triple negative breast cancer, lower expression of ASCT2 was correlated with better survival in both patients and genetically modified mouse models [23].

The understanding of glutamine uptake has led to the development and application of new imaging modalities for cancer diagnosis. Traditionally, ^18^F-flurodeoxyglucose (^18^F-FDG) coupled with positron emission tomography (PET) has been used for imaging tumors due to increased glucose uptake but has some tissue specific limitations including gliomas due to high background uptake in the brain [27]. Studies identifying increased expression of glutamine importers and biochemical evidence of increased uptake have led to PET imaging with glutamine analogs such as 4-^18^F-(2S,4R)-fluoroglutamine (^18^F-FGln) [27]. Subsequent studies in human patients have reported the potential application for clinical imaging as ^18^F-FGln highlighted tumors from various tissues (including breast, pancreas, lung, and colon), which corresponded with altered glutamine metabolism [28]. As mechanistic understanding of glutamine transport and utilization continues to expand, it is certain that new applications will develop [29].

## 3. Glutamine in Protein Synthesis

As a proteogenic amino acid, glutamine serves an essential component in the synthesis of proteins, with decreases in glutamine levels being correlated to decreases in protein synthesis [30]. This is not unexpected as deficiency of any amino acid alters amino-acylation of tRNAs, which in turn lead to GCN2 activation, with subsequent phosphorylation of eIF2α on Serine 51. This phosphorylation event leads to a global pausing of translation [31,32]. Glutamine can also indirectly support protein synthesis by contributing its carbons to the synthesis of other amino acids (glutamate, asparagine, aspartate, and proline) [33]. Cells can overcome glutamine deficiency through the use of asparagine for proliferation and protein synthesis. Interestingly, the exogenous addition of asparagine on glutamine-deprived cells increases protein levels of glutamine synthetase (GS), the enzyme responsible for de novo synthesis of glutamine, and subsequently enhances incorporation of glucose carbons into glutamine. Asparagine could not enhance cell growth and protein synthesis when GS was genetically ablated or pharmacologically inhibited [34]. 

## 4. Catabolism and Anabolism of Glutamine

The emphasis on glutamine metabolism in cancer has been focused on glutamine catabolism. The first step of glutamine utilization is its conversion to glutamate, which can be catalyzed by enzymes in the cytosol or mitochondria [26,35]. In mitochondria, glutamine can be catabolized by glutaminase (GLS) via hydrolytic cleavage of glutamine which results in glutamate and an ammonium ion [36].

GLS is encoded by two genes producing two proteins, each with multiple isoforms as a consequence of alternative splicing. They differ in their tissue-specific activities and kinetics; GLS1 has high glutaminase activity and low Km, while GLS2 has low activity and high Km [37]. GLS1 is the kidney type glutaminase, expressed predominately in the kidney and brain. GLS1 has both a long and short isoform which exhibit differences in the C-terminus, although both isoforms are reported to be localized to mitochondria [38]. GLS1 has generated significant interest in the cancer field, due to its regulation by c-Myc [39]. c-Myc was shown to increase expression of mitochondrial glutaminase (GLS1) which was necessary for proliferation and survival. This Myc-driven GLS1 expression is not through direct binding of Myc to the GLS1 gene, but rather through Myc-mediated suppression of micro-RNAs miR-23a and miR-23b that could target the 3′ UTR of GLS1 leading to decreased levels of the enzyme [39]. 

When extracellular supply of glutamine is scarce, cells can upregulate their autonomous production of glutamine, which is mediated by GS, otherwise known as glutamate-ammonia ligase (GLUL). The human GLUL gene is located on chromosome 1q23. It is the only enzyme capable of de novo glutamine synthesis which catalyzes the condensation of glutamate and ammonia in an ATP-dependent manner [40]. This reaction is unique as the use of free ammonia in the majority of metabolic pathways, such as amino acid and nucleotide biosynthesis, is insignificant. Cells prefer utilizing amino acids to transfer nitrogen between molecules [41]. GS plays a role in nitrogen metabolism, ammonia detoxification, and cell signaling [42]. It is commonly elevated in cancers and has been demonstrated to have a role in polarizing macrophages from an M2 to M1-like phenotype [43,44,45,46]. This cell autonomous production of glutamine is intriguing, particularly in cancer, which has been suggested to convey independence from extracellular glutamine levels [44,45,46,47]. 

Recently, the structure of GS has been solved. Human GS is a decamer, a set of two identical pentamers which interact to form a “funnel” shape structure. The full protein has ten active sites that are situated at the interface of adjacent subunits in the pentamer ring [48]. The protein is conserved from bacteria to human, albeit with low sequence similarity. Regardless, the structural conformation of the active site is conserved. The bacterial enzyme contains a longer C-terminus with a critical tyrosine residue that serves as a site for protein adenylation to modulate activity [48,49]. GS levels and activity can be altered by the availability of substrates and products. This phenomenon has been long appreciated and well characterized in bacteria [49,50,51]. A recent study showed that eukaryotic GS is acetylated at two lysine residues in response to elevated levels of glutamine, and these acetylation modifications serve a role in ubiquitination by CRL4^CRBN^, an E3 ligase [52], which eventually leads to GS degradation via proteasomes.

## 5. Metabolic Fate of Glutamine

An abundance of studies has provided biochemical evidence with stable isotope tracing that help determine the exact fate of glutamine carbon and nitrogen [26,33,35,44,45,53]. Glutamine as a carbon source has long been appreciated. It can serve as an anaplerotic substrate for the TCA cycle contributing in rapidly proliferating or neoplastic tissues, especially in the context of Myc-expressing cells [19]. A study showed that lack of glutamine could induce apoptosis in a Myc-dependent manner. Cell death followed the depletion of TCA intermediates, although interestingly was unrelated to the ATP level. Cell death could be rescued with the addition of pyruvate or oxaloacetate, which led to the conclusion that the carbon aspect of glutamine was critical [54]. A concurrent study suggested that tumor cells use glutamine at an elevated rate, which does not simply correspond to the need for biosynthetic pathways. These transformed cells undergo aerobic glycolysis and produce lactate, but still use glucose carbons in the TCA cycle which could exit the cycle in the form of citrate for fatty acid synthesis. In these cells, glutamine was catabolized to α-ketoglutarate and entered the TCA cycle to maintain a carbon threshold in the TCA cycle and manage NADPH redox status [35]. A subsequent study interrogated the connection between c-Myc and glutamine utilization [26]. The initial findings were a logical extension from previous studies [54], such that glutamine carbons were highly used in tumor cells albeit with a minor fraction being incorporated into proteins. It was shown that deprivation of glutamine induced cell death and that α-ketoglutarate supplementation could mitigate this phenotype. Modulation of c-Myc resulted in changes in levels of downstream transcriptional targets that were involved in glutamine uptake and metabolism, such as ASCT2, and that c-Myc hyperactivation sensitizes cells to glutamine starvation [26].

There is a long-standing appreciation that glucose and glutamine are avidly consumed by proliferating mammalian cells and are the most highly consumed constituents of media, but the exact contribution of these molecules to cell mass including proteins, amino acids, and polar metabolites is unknown. Surprisingly, both nutrients contribute minor amounts of carbon to cell mass, with glutamine contributing less than 10% of total cell mass. Instead, a pooled mixture of 15 essential and non-essential amino acids (not including glutamine) labeled the majority of cellular carbon. This is consistent among cells from various tissues and oncogenic drivers. Quantitative measurements were also made using either amide- or alpha-labeled glutamine, which resulted in almost 30% of the total cellular nitrogen pool [33]. 

Glutamine-derived glutamate can serve as a substrate for multiple enzymes, including transaminases and glutamate dehydrogenase (GLUD), sending the carbon backbone towards TCA anaplerosis. Catabolism of glutamate via GLUD1 results in ammonia being released in mitochondria. Transaminases exist in the cell that function to transfer the amino group of glutamate to keto-acids in order to synthesize amino acids, such as glutamic oxaloacetic transaminase (GOT) and glutamate-pyruvate transaminase (GPT). GOT mediates the transfer of nitrogen from glutamate to oxaloacetate, resulting in alpha-ketoglutarate and aspartate. GPT mediates the transfer of nitrogen from glutamate to pyruvate, resulting in alpha-ketoglutarate and alanine. By way of these enzymatic reactions, the 5-carbon backbone of glutamine/glutamate is converted to alpha-ketoglutarate which can enter the TCA cycle or serve other roles such as acting as a cofactor for epigenetic remodeling enzymes [55]. By way of the same reactions, nitrogen can be dispersed into the cell to function in multiple pathways including shuttling metabolites between the mitochondria and cytosol, contributing to redox regulation, or serving as fuel in non-canonical ways [56,57].

The amide component of glutamine can be used in a number of ways, most predominantly for the biosynthesis of amino acids, nucleotides nicotinamide-adenine dinucleotide (NAD) and hexosamines. As mentioned earlier, the deamidation of glutamine by glutaminase leads to the release of ammonium ion. While ammonia in the cell is potentially toxic, a recent study has suggested ammonia can be assimilated by GLUD1 to generate amino acids, such as aspartate proline and leucine, a reaction running in reverse with ammonia liberated from glutamine via GLS [58]. The amido group of glutamine is also required for the synthesis of asparagine. Asparagine synthetase catalyzes the production of asparagine and glutamate from aspartate and glutamine in an ATP-dependent amidotransferase reaction [18,59].

Nucleotide biosynthesis is critical for cellular function. Nucleotides are required for replication of the genome and transcription of DNA into RNA. The nucleotide biosynthesis pathway is also an important intervention point for cancer therapeutics. In fact, nucleoside analogues (gemcitabine and 5FU) and antifolate (methotrexate) are the standard chemotherapy treatments for a variety of cancers, including pancreatic, breast, lung and head and neck cancers and certain types of lymphoma, and leukemia [60].

Mammalian cells have two pathways for acquiring nucleotides, the salvage pathway and de novo synthesis. De novo production of nucleotides is an energy demanding process, which consumes glutamine and aspartate molecules to contribute to the purine or pyrimidine ring [61]. During purine synthesis, γ (amido) nitrogens from two glutamines and one α (amino) nitrogen from aspartate are needed. In pyrimidine synthesis, processing of glutamine by cytosolic amido-transferase, carbamoyl phosphate synthase II (CPSII), transfers the γ nitrogen from glutamine to bicarbonate in order to produce carbamoyl phosphate, the initiating metabolite in pyrimidine synthesis [61,62]. Three carbon atoms and an amino nitrogen from aspartate are also required in pyrimidine biosynthesis [61]. As aspartate can be derived from glutamine via the TCA cycle and transamination, glutamine can also contribute aspartate to the pathway.

Underlining the importance of glutamine in nucleotide biosynthesis, studies have repeatedly shown that cancer cells under glutamine deprivation undergo DNA damage response and cell cycle arrest. The phenotype can be rescued by the addition of nucleotides [63]. Several studies have also emerged demonstrating that the increase in de novo glutamine synthesis via GS in cancer cells and animal models fuels nucleotide biosynthesis and consequently promotes cancer cell growth [44,45,64].

NAD is a co-enzyme that mediates redox reactions in several metabolic pathways, such as TCA cycle and oxidative phosphorylation. Various studies have demonstrated NAD and enzymes important in the biosynthesis of the co-enzyme can support survival of cancer cells by modulating pathways like energy metabolism, gene transcription and cellular stress response [65]. NAD is synthesized via the salvage pathway or de novo pathway. De novo NAD biosynthesis requires multiple amino acids. The first step of NAD synthesis requires tryptophan conversion to N-formylkynurenine. The terminal step requires glutamine as a nitrogen donor in the amidation of nicotinic acid adenine dinucleotide to NAD in an ATP dependent reaction [66].

Another important metabolic fate of glutamine amide is the biosynthesis of hexosamines. Divergence of carbons from the glycolytic pathway in the form of fructose-6-phosphate results in the production of amino sugars (hexosamines) which can be used for protein modification. The hexosamine biosynthesis pathway (HBP) is a nexus between glucose and glutamine in the cell [67]. The first rate-limiting step of the HBP is the conversion of fructose-6-phosphate to glucosamine-6-phosphate which is catalyzed by glutamine fructose-6-phosphate aminotransferase 1 (GFAT1). GFAT1 transfers the amide nitrogen of glutamine to fructose-6-phosphate and subsequently receives an acetyl group from acetyl-CoA, via a glucosamine-6-phosphate acetyltransferase [68], to form N-acetyl-glucosamine-6-phosphate (GlcNAc-6-P) and undergoes an isomerization reaction to N-acetyl-glucosamine-1-phosphate (GlcNAc-1-P). Uridine diphosphate-GlcNAc pyrophosphorylase catalyzes the ultimate step in the pathway, consuming GlcNAc-1-P and uridine triphosphate (UTP) to produce UDP-GlcNAc [67]. 

UDP-GlcNAc is used for O-linked-β-N-acetyl glucosamine modification (O-GlcNAcylation), a protein modification in which GlcNAc is conjugated to serine and threonine residues of proteins. Production of UDP-GlcNAc and O-GlcNAcylation are emerging as a means to link nutrient levels to intracellular functions [67,69,70], and has been implicated in regulating intracellular signaling which impact tumorigenesis [71,72]. 

## 6. Disease

Relatively little is known about inborn errors of metabolism that pertain to glutamine. GS is essential during embryonic development, especially in the blastocyst stage. Its germline deletion is embryonic lethal in mice at day E2.5. GS-knockout embryos can be grown in vitro by supplementation with glutamine, with no observable growth or morphological defects. Complete deficiency in GS in human patients has not been reported in the literature, although rare patient cases have been identified with reduced enzyme expression or activity [42]. Two patients were identified with congenital systemic GS deficiency. Both patients exhibited a mutation in the coding region of GS, R324C and R341C, which resulted in decreased enzyme activity. Both children were born to consanguineous Turkish parents, died within the first month of life due to brain malformation which resulted in multiorgan failure. Both patients exhibited normal glutamate levels in the serum, albeit glutamine levels were significantly reduced [42]. Recently, an additional patient has been identified, a Sudanese boy born to consanguineous parents with a mutation (R324S) and reduced glutamine levels in serum and urine, although higher than the originally identified patients [73]. The arginine residue (R324) is located in the catalytic site and is involved in the binding of ATP. R341is thought to be involved in the binding of glutamate to glutamine synthetase [48,73]. Interestingly, patient data suggests that reduced activity of glutamine synthetase results in NAD depletion [74]. Supporting the idea that glutamine is important in NAD biosynthesis, fibroblasts extracted from the patient carrying the GLUL gene point mutation showed that the marked depletion of NAD can be rescued by supplementation of glutamine, nicotinate or nicotinamide. Taken together with evidence from animal models and human patient data, GS plays an essential role in embryonic development.

A recent study demonstrated a novel expansion of the trinucleotide tandem-repeat in the 5′ untranslated region (5′UTR) of the GLS gene in three unrelated patients. These patients presented with early-onset delay in both gross and fine motor skills, as well as delayed speech. Biochemical studies of patients found elevated levels of glutamine and a decrease in glutamate levels due to reduced levels of GLS expression and activity. All three patients developed ataxia, a phenomenon also seen in patients with other causes of glutamate deficiency [75]. In two of the patients, exome analysis revealed a maternally or paternally inherited heterozygous GLS variant. What was consistent in all patients was the expansion of GCA in the 5′ untranslated region in at least one allele. This expansion lead to reduced levels of histone H3 acetylation and H3K4 trimethylation modifications that enable chromatin regions permissive for transcription. Patients were also enriched for a histone H3K9me3, which suppresses transcription. Altogether, the study identified a novel mechanism on how non-coding regions (GCA-repeat tract on 5′ UTR) of the genome, that are usually neglected in the clinic due to preferred usage of exome sequencing for molecular diagnosis of patients, can dictate binding of histones and subsequently determine transcription of a particular gene [76]. Most importantly, this study emphasizes the importance of glutaminase in human development, in the context of the brain and nervous system. 

The clinical studies mentioned above underline the importance of glutamate/glutamine homeostasis and availability in vivo. Nutrient availability in the context of disease has long been appreciated. Nutrient shortage in cancer is a physiological feature of certain tumors and environments, but may yield actionable approaches. Reports in the literature have identified disease types which are auxotrophs for certain amino acids, such as asparagine auxotrophs in acute lymphocytic leukemia or arginine auxotrophs in clear cell renal carcinoma [22,77,78]. The majority of reports pertaining to glutamine indicate increased expression of enzymes, perhaps indicating that glutamine auxotrophs are disadvantaged.

## 7. Glutamine Addiction is Context-Dependent in Cancer

The long-standing dogma in cancer biology, is the enhanced dependency of most cells on glutamine, often termed “glutamine addiction” [19,26,79]. Findings from recent studies imply that the environment, at least in part, dictates glutamine usage. A major expansion of the literature pertaining to glutamine metabolism in cancer has begun to rewrite that paradigm and convey the notion that glutamine metabolism in cancer is highly heterogeneous. Recently it has been highlighted that tissue of origin can be a major determinant in metabolism of the tumor [80]. For instance, mouse models of tumorigenesis expressing c-Myc in various tissues, results in expression of different metabolic enzymes including lactate dehydrogenase, GLS1, GLS2, and GS [81]. The same study demonstrated that the genetic lesion of the tumor is also a determinant of the metabolic preference of tumors; Myc-induced mouse liver tumors increased glutamine and glucose catabolism, while MET-induced liver tumors used glucose to generate glutamine [81]. This is further complicated by evidence that suggests cells in vivo use certain metabolites as fuel, but this is altered when the cells are taken ex vivo and grown in culture [53]. Subsequent studies have suggested that this metabolic shift is an effect of non-physiological levels of nutrients [82,83]. For example, sensitivity to glutaminase inhibition was shown to be driven in part by high levels of exogenous cystine in cell culture media, which is involved in the transport of glutamate via the antiporter xCT/SLC7A11 [82]. 

Emerging evidence highlights the variance of metabolism, specifically as it relates to glutamine, in vitro as compared to in vivo. Glutamine catabolism was found to be negligible in multiple Kras-driven non-small cell lung cancer (NSCLC) [53]. The conversion of ^13^C-glutamine to ^13^C glutamate was minimal in normal lung tissue as well as various tumor models, indicating that canonical consumption of glutamine was not actively occurring at a substantial rate in vivo. Perhaps most interestingly, when cell lines were derived from Kras/TP53 driven tumors, which did not exhibit significant glutamine catabolism in vivo, they acquired a dependency on glutamine in the media and sensitivity to glutaminase inhibition in 2D and 3D cultures. Transplantation of these cells into the lungs of syngeneic mice resulted in a metabolic phenotype similar to spontaneous tumors. These data indicate that the environment plays a critical role in dictating the metabolism of the tumor [53]. These data are in agreement with the study that demonstrated pyruvate carboxylase (PC) was upregulated in NSCLC, while GLS1 levels were not elevated. NSCLC patients were infused with ^13^C-glucose which revealed PC was active in vivo as evidenced by levels of ^13^C-aspartate and ^13^C-citrate. Subsequent ex vivo incubation of cancerous and noncancerous patient samples in ^13^C,^15^N-glutamine revealed that glutaminase was active, but the enrichment pattern of isotopologues was comparable between both sets of tissues leading to the conclusion that glutaminase is active but not increased in NSCLC [84]. Another recent study infused ^13^C-glucose into mice bearing primary glioblastoma samples. Comparing the implanted tumors to the surrounding brain revealed that glioblastoma cells engaged in mitochondrial glucose oxidation. Compared to the surrounding normal brain, these tumors had reduced levels of GLS1 expression. Analysis of glucose tracing indicated that the tumors engaged PC for anaplerosis and in fact converted some glucose derived carbons into glutamine via glutamine synthetase [85]. Together, these studies indicate a context-dependent impact on glutamine metabolism and addiction which extends beyond GLS-mediated glutamine catabolism.

## 8. Glutamine Metabolism in the Tumor Microenvironment

**Figure 2:**
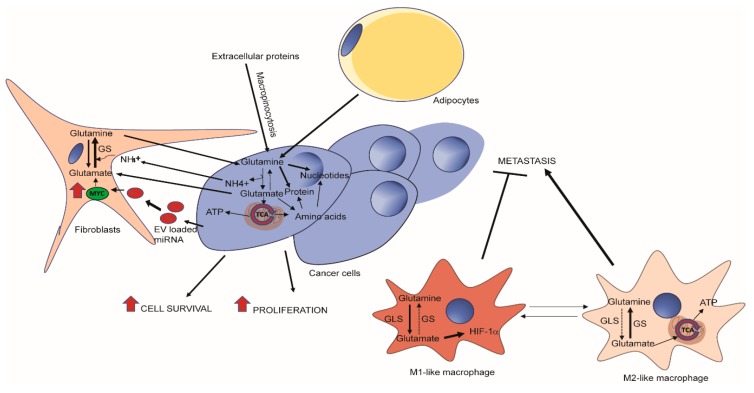
Glutamine metabolism in tumor microenvironment. Tumor cells can obtain glutamine via macropinocytosis of extracellular proteins, or via the import of free glutamine secreted by cancer-associated fibroblasts (CAFs) or adipocytes [47,92]. Tumor cells communicate with CAFs via instructional miRNAs in exosomal vesicles (EVs) and secrets glutamate and ammonia as wastes that can be taken up by CAFs for glutamine synthesis [93]. In a cell autonomous manner, a decrease in glutamine synthetase activity in tumor-associated macrophages (TAMs) can reprogram glucose and glutamine metabolism and lead to polarizing cells from an M2 to M1-like phenotype which inhibits metastasis [43].

The tumor microenvironment presents a complex system for metabolic exchange and crosstalk (Figure 2). In pancreatic cancer, it is appreciated that the bulk of the neoplasia is in fact not cancer cells, but fibrotic cells and immune cells [86,87]. This desmoplasia can act as a barrier which prevents the delivery of chemotherapeutic drugs but also limits the supply of blood and in turn oxygen and nutrients [88]. Pancreatic cancer cells have been shown to exhibit “non-canonical” glutamine metabolism, characterized by utilization of the glutamine catabolic enzyme GOT1, culminating in the increase of NADPH/NADP+ ratio to maintain redox status [56]. PDAC cells have been shown to make use of alanine secreted from the surrounding pancreatic stellate cells, which form a major component of the tumor [57]. Adipocytes may also play a role in supplementing pancreatic ductal adenocarcinoma (PDAC) cells with glutamine in nutrient-deprived PDAC environment. In fact, peri-tumor adipocytes predict poor prognosis in PDAC [89,90]. Adipocytes have been shown to secrete glutamine in their conditioned media and therefore may act to support cellular growth of glutamine-deprived pancreatic cancer cells [91].

This metabolic exchange is not limited to pancreatic cancer. An increasing number of studies emphasize the role of tumor niche in promoting cancer cell growth through metabolic crosstalk in a variety of cancers, such as breast cancer, glioblastoma, and ovarian cancer [44,45,47,92]. Indeed, a study on tumor-stroma interaction in ovarian cancer has implicated glutamine metabolism as a complex exchange between the different cell populations. It has been shown that glutamine anabolic pathways are upregulated in cancer associated fibroblasts (CAFs) from advanced stage, high-grade ovarian adenocarcinomas, when compared to normal ovarian fibroblasts (NOFs). This includes GS as well as amino acid transferases such as GOT1/2 and BCAT1 which corresponded with the ability of CAFs to synthesize glutamine from a variety of fuel sources, and allowed CAFs, but not NOFs, to support cancer cell growth under glutamine deficiency. Mechanistically, cancer cells secrete glutamate and lactate which are acquired by CAFs and used to generate glutamine via GS, which is subsequently secreted for uptake by cancer cells for glutaminolysis and nucleotide production. Silencing of stromal GS and tumoral GLS led to a synthetic lethality [47]. Adding complexity to the metabolic cross-talk between cancer cells and CAFs, a recent study demonstrated breast cancer-cell-secreted exosomal miR-105 reprogrammed CAF metabolism in a Myc-dependent manner to enhance metabolic symbiosis between the cancer cells and niche cells. In a nutrient replete environment, treatment of CAFs with extracellular vesicle-encapsulated miR-105 increased glutamine and glucose catabolism, secreting glutamate, acetate, and lactate, to feed adjacent cancer cells. In nutrient-depleted conditions, cancer-secreted miR-105 increased expression of enzymes important in gluconeogenesis and glutamine anabolism in CAFs to consume and detoxify extracellular ammonium and lactate released by cancer cells. The process is important for the conversion of these metabolites into non-toxic components that can be reused for bioenergetic pathways in cancer cells [93].

Glutamine metabolism plays an important role in tumor-associated macrophages, resident innate immune cells that can regulate angiogenesis and T-cell activation and recruitment in tumors. A recent study has shown that pharmacological inhibition or genetic ablation of glutamine synthetase in macrophages promotes an M1-like phenotype. Intracellular glutamine levels were reduced and succinate levels increased with enhanced glycolysis and subsequent activation of HIF1α. These changes lead to an increase in T cell recruitment and suppression of proangiogenic state and metastasis in a mouse model where Lewis lung carcinoma (LLC) cancer cells were subcutaneously implanted [43]. 

## 9. Therapeutics

A number of inhibitors for GLS have been developed and showed remarkable efficacy in vitro. 

Bis-2-(5-phenylacetamido-1,2,4-thiadiazol-2-yl)ethyl sulfide (BPTES) is specific for the kidney glutaminase isoform (GLS1) [36]. Glioma cells harboring a mutant form of isocitrate dehydrogenase (IDH1) exhibited sensitivity to BPTES treatment, which displayed minimal effects on the wildtype IDH1 protein [94]. An additional glutaminase inhibitor, compound 968, was shown to block oncogenic transformation in fibroblasts, while also reducing growth of cancer cells without affecting their normal counterparts [95]. A glutaminase inhibitor which targets both kidney and liver splice variants of glutaminase (CB-839) has been shown to be selective and effective in vitro and is currently undergoing clinical trials [96]. In triple-negative breast cancer, CB-839 reduced glutamine consumption, glutamate production, and levels of TCA intermediates, all of which contributed to the antiproliferative activity [97]. Results in vivo have demonstrated that the glutaminase activity is maintained upon acquisition by tumors, but the antitumor capacity can be lacking [53]. It is possible that the environment and metabolic milieu that accompanies the tumor is responsible for the level of sensitivity to glutaminase inhibition [53,98]. Metabolic plasticity of cancer cells can also contribute to the efficacy of these inhibitors. A recent study showed pancreatic cancer cells rewire their metabolic needs after pharmacological inhibition of GLS to maintain proliferation in vitro and in vivo. Withdrawing PDAC cells of one of their favored carbon source, glutamine, leads to attempts by the cell to acquire carbon from alternative pathways, such as oxidation of branched chain fatty acids [99].

Methionine sulfoximine (MSO) is an irreversible inhibitor of GS, which functions by binding in the glutamate active site, and is subsequently phosphorylated [100]. MSO usage in the biotechnology industry for production of monoclonal antibodies is common, acting as a selection tool via overexpression of glutamine synthetase in conjunction with a gene of interest, then selected for with MSO [101]. Cancer cells treated in vitro with MSO showed reduced growth, decreased survival, and decreased glutamine synthetase activity which correlated with decreased de novo synthesis of glutamine and downstream nitrogen metabolites such as nucleotides [44,45]. Mice treated intraperitoneally with MSO displayed no adverse effects [102,103]. Growth of xenografts of human hepatocellular carcinoma cells could be inhibited with MSO treatment. Animals cotreated with MSO and bacterial asparaginase, which also depletes circulating glutamine, yielded a greater reduction in tumor growth [104].

6-Diazo-5-oxo-L-norleucine (DON) is a glutamine antagonist originally isolated from *Streptomyces* and was demonstrated to have antitumor properties [105]. DON acts as a rather promiscuous inhibitor, residing in glutamine binding sites of various enzymes [105]. Upon competitive binding, a covalent adduct is created which leads to irreversible inhibition [106]. DON is capable of inhibiting mitochondrial GLS, GS, numerous enzymes in the nucleotide synthetic pathway, and asparagine synthetase [105,106]. Due to the multitude of targets, DON can be efficacious in the treatment of disease, albeit with potentially strong side effects. Efforts to eliminate side effects are ongoing, including development of prodrugs which exhibit minimal release of DON to gastrointestinal tissues or increased release of DON in tumor cells [106,107]. The wide spectrum of enzymes inhibited by DON leads to tempting speculation of disease treatment when the complex metabolic crosstalk of tumor and stroma is taken into consideration. A single agent therapy which could inhibit both glutamine synthesis, as well as all avenues of glutamine consumption merits further investigation.

## 10. Future Perspectives

Glutamine is a pleiotropic molecule that plays a role in many cellular processes, from protein synthesis to nucleotide production, from mediating uptake of other amino acids to transporting ammonia from tissue to tissue. The ability to synthesize glutamine is essential for development but is a process that can be hijacked for oncogenesis, especially under glutamine-limitation conditions. The increased demand for macromolecule production, energy production, and biomass are cornerstones of cancer growth. It is important to consider both the anabolism and catabolism of glutamine in the context of disease.

Studies have mainly focused on targeting the catabolism of glutamine in cancer, but recent research have demonstrated the importance of glutamine in protein synthesis and amino acid exchange and its amido group in the biosynthesis of hexosamine, nucleotides, asparagine and NAD. These pleiotropic effects of glutamine are crucial for tumorigenesis and occur independent of glutamine catabolism. Therefore, simultaneously targeting the uptake of glutamine and de novo glutamine synthesis in both cancer cells and tumor associated cells (that can supply glutamine to cancer cells) must be strongly considered as a therapeutic approach. However, in vivo models have shown glutamine usage is dependent on genetic lesion of tumor and tissue of origin [53,80,81]. Therefore, further studies are needed to determine what types of patients will benefit from therapies targeting glutamine metabolism. We should also keep in mind that glutamate is the major excitatory neurotransmitter in the brain [108] Animal models have repeatedly shown the importance of glutamine-glutamate cycle during glutamatergic synaptic transmission [109]. As demonstrated earlier, GLS and GLUL deficiency in humans, negatively impact brain development and motor skills (e.g., ataxia) [42,73,76]. Therefore, we should strongly consider the possible symptoms patients might experience if GLS and GLUL inhibitors that can cross the blood-brain barrier are used in the clinic for cancer therapy. 

While glutamine has been extensively studied for its roles in both normal physiology and cancer, new evidence is expanding our knowledge of its multitude of functions. As new technologies emerge, the field will gain the capacity to probe metabolism in vivo with greater resolution to understand the spatial and temporal changes that occur during normal tissue function or on the path to tumorigenesis. This heightened understanding of metabolic function as it occurs in the body will inform drug development and therapies to make the leap from the bench to the clinic.
